# Juglanin Inhibits Osteoclastogenesis in Ovariectomized Mice via the Suppression of NF-κB Signaling Pathways

**DOI:** 10.3389/fphar.2020.596230

**Published:** 2021-01-27

**Authors:** Fangxue Zhang, Xiaowei Huang, Yuhan Qi, Zhi Qian, Shuo Ni, Zeyuan Zhong, Xu Zhang, Dejian Li, Baoqing Yu

**Affiliations:** ^1^ Department of Orthopedics, Shanghai Pudong Hospital, Fudan University Pudong Medical Center, Shanghai, China; ^2^ Department of Orthopedics, The First Affiliated Hospital of Soochow University, Suzhou, China; ^3^ Department of Plastic Surgery, First Affiliated Hospital of Nanchang University, Nanchang, China; ^4^ Department of Oral & Cranio-Maxillofacial Surgery, Shanghai Ninth People's Hospital, College of Stomatology, Shanghai Jiao Tong University School of Medicine, National Clinical Research Center for Oral Diseases, Shanghai Key Laboratory of Stomatology & Shanghai Research Institute of Stomatology, Shanghai, China

**Keywords:** juglanin, osteoclastogenesis, NF-κB pathways, ovariectomized-mice, RANKL

## Abstract

Bone metabolism is a physiological process that involves both osteoblasts and osteoclasts. Pathological changes of osteoclasts are commonly seen in osteoporosis diseases. Juglanin is a natural compound, reported to have an inhibitory effect on inflammation, oxidative stress and cancer progression. The purpose of this study is to explore the role that Juglanin plays on the osteoclast functions and underlying signaling pathways. *In vitro* study demonstrated that Juglanin had negative influence on osteoclastic differentiation by suppressing the transcription activity of osteoclastogenesis-related genes and proteins. To determine the underlying mechanism, Western blot was employed to show that Juglanin could significantly have negative effect on the phosphorylation of P50, P65, I-κB， ultimately suppressing the expression and transcriptional activity of nuclear factor of activated T cells (NFATc1). *In vivo* Juglanin treatment attenuate bone reducing in mice with removed ovary through suppressing osteoclast functioning. Taken together, our study demonstrated that in the molecular mechanism, JUG inhibited the expression of receptor activator of nuclear factor-κ B ligand (RANKL) induced NF - *κ* B signaling pathway, thus may play a vital part in preventing postmenopausal osteoporosis.

## Introduction

Osteoporosis is the disease with high incidence rate which is manifested by bone loss and bone microstructure reducing, resulting in impaired rigidity as well as increased risk of fracture (Ensrud and Crandall, 2017; Leutner et al., 2019). Postmenopausal osteoporosis (PMO) is a typical orthopedic disease caused by excessive activation of osteoclasts (Ensrud and Crandall, 2019). Stepan et al. (Curr Osteoporos Rep, 2019) reported that estrogen can play an anti-osteoporotic effect by promoting the secretion of osteoprotegerin (OPG) in osteoblast and inhibit the formation of osteoclasts (Allison and McNamara, 2019; Stepan et al., 2019). On the other side, it was also observed that the osteoclastogeneis process was over-activated after menopause, thus leading to net bone loss and increased risk of osteoporosis (Levin et al., 2018; Kanis et al., 2019).

Osteoclast belongs to the monocyte-macrophage family and is the key member in bone metabolism (Madel et al., 2019). Several cytokines are responsible for osteoclastogenesis (Lorenzo, 2017). RANK (NF-κB receptor activator) and its ligand RANKL initiate the signaling for osteoclast formation (Park et al., 2017; Choi et al., 2018; Funakubo et al., 2018; Ikebuchi et al., 2018), recruiting TNF receptor related factors 6 which further stimulate the phosphorylation of down-stream transcription factors (Sambandam et al., 2016; Wu et al., 2016; van Dam et al., 2019). It has been shown that selective inhibition of NF-κB and MAPK pathways can reduce osteoclast formation (Yao et al., 2017; Kim et al., 2018).

Juglanin (JUG) is a natural compound derived from the *crude Polygonum aviculare*. It has been reported the inhibitory effect on inflammation, oxidative stress and cancer progression. According to previous studies, juglanin prevents hepatitis through inflammation suppression by inactivating TLR4/NF-κB signaling pathway (Chen et al., 2017; Sun et al., 2017; Hou et al., 2018; Zhang and Xu, 2018; Chen et al., 2019). In addition, JUG has been explored in the occurrence of human breast cancer through apoptosis, by inducing reactive oxygen species (ROS) production in cells (Sun et al., 2017). However, the effect of JUG on osteoclastogenesis remains unknown. Therefore, in our research, we studied the role JUG play in the process of osteoclastogenesis, and the potential mechanism of JUG on RANKL-treated osteoclasts. In addition, a mouse model of bone loss was built by ovariectomy (OVX) to validate its effectiveness *in vivo*.

## Material and Methods

### Reagents and Antibodies

The JUG with a purity of more than 98% was obtained from Nancheng biochemistry (Shanghai, China) and dissolved in DMSO as for storing with concentration of 1 μmol/L and stored at −20°C. Further dilution was carried out in culture medium for cells and PBS medium for animals. Primary antibodies against NFATc1 (#8032, CST) and V-ATPase-d2 (#ABS1677, Sigma) were acquired from Zhongshantech (Wuhan, China). Primary antibodies for P50 (#13586, CST), P-P50 (#4806, CST), P65 (#4764S, CST), P-P65 (#3033, CST), and IκB (9242, CST), P- IκB (#2859, CST) were delivered by Cell Signaling biology (TA, United States). MMT kit was bought from Tengyi Technology (Nantong, China).

### Cell Culture, Cell Viability Assay and Osteoclastogenesis Assay

As mentioned in previous studies (Sambandam et al., 2016; Wu et al., 2016; van Dam et al., 2019), in order to extract bone marrow mononuclear cells (BMM), C57BL/6 mice between 4 to 6 week-old were executed and the lower limbs were dissected. 1 ml syringe was used to wash the cells out of the bone cavity of femoral and tibia. The cells were then co-cultured by 30 ng/ml M-CSF (Macrophage Colony stimulating Factor) for 24 h. Removing the non-adherent cells, the attached cells were kept until the cells reached 80% of confluency. For cell viability determination, BMMs were plated on 96-well plates with a concentration of 10,000 cells/well. MTT assay was then performed to detect JUG cytotoxicity. After a 24-h incubation, different concentrations of JUG (0, 10, 20, 40, 80, 160, 320, 640 μmol/L) was then added and cultured with the cells for 72 h (Chen et al., 2018b; Maridas et al., 2018). In order to differentiate osteoclasts, the cells were stimulated with 30 ng/mL M-CSF, 50 ng/ml RANKL and different concentrations of JUG (0, 20, 40 or 80 μmol/L). Changing the medium regularly until osteoclasts formed and matured and fixing the cells with 4% paraformaldehyde for 20 min, then the staining process was conducted. The stained cells with no less than 3 nuclei were scored.

### F-Actin Ring Formation Assay

RAW 246.7 cells were stimulated with JUG of different doses for 5 days as well as 50 ng/ml RANKL. Then a 0.25 percent Triton X-100 was used to penetrate the cell membrane. The cells were blocked in 3% BSA. After blocking, the f-actin ring was labeled with Rhodamine coupled phalloidin (Eugene, Oregon, USA), and the nucleus of osteoclasts was labeled with DAPI (Chen et al., 2018a).

### Determination of Absorption Pit

Bone resorption is the most important function of osteoclasts. The test of osteoclast absorption function is the gold standard of osteoclast examination. In this study, the absorption pit assay was used to evaluate the bone resorption function. BMMs were plated into 6-well plates at a density of 8 × 10 ^4 cells/well and cultured with 30 ng/ml M-CSF for 3 days, then stimulated with 30 ng/ml M-CSF and 50 ng/ml RANKL for 5 days until osteoclast formation. Osteoclasts were then implanted into 96-well plates, each with bone slices. After co-culture with osteoclasts for 48 h, hematoxylin staining was performed to detect the absorption pit (Chen et al., 2018b).

### Immunofluorescence Staining

Immunofluorescence was used to evaluate the nuclear translocation of p65. Briefly, BMMs were fixed with 4% PFA, then, washing with Triton X-100 to facilitate staining, followed by incubated with anti-p65 antibody, goat anti-mouse IgG antibody, the result was observed by microscope.

### Real-Time PCR

Real-time quantitative polymerase chain reaction (qRT-PCR) is used to quantify the mRNA expression of osteoclastogenesis related genes. Using TRIzol reagent, the total RNA of RAW 264.7 was extracted in a 6-well plate, followed by reversely transcribing to cDNA. The sequences of the primers used are: cathepsin K(CtsK) (forward: 5-GGG​AGA​AAA​ACC​TGA​AGC-3’; reverse: 5′-ATTCTGGGGACTCAGAGC-3′); c-Fos (forward: 5′- GCGAGCAACTGAGAAGAC - 3′, reverse: 5′- TTG​AAA​CCC​GAG​AAC​ATC-3′); TRAcP(forward: 5′-TGT​GGC​CAT​CTT​TAT​GCT-3’; reverse:5′GTCATTTCTTTGGGGCTT-3′); MMP-9 (forward: AGT​TTG​GTG​TCG​CGG​AGC​AC; reverse: TAC​ATG​AGC​GCT​TCC​GGC​AC), GAPDH (forward: AAC​TTT​GGC​ATT​GTG​GAA​GG; reverse:ACACATTGGGGGTAGGAACA). The parameters of RT-PCR was set according to previously published papers (Tan et al., 2017).

### Western Blot Analysis

Cells were lyzed to obtain total protein content in freshly prepared frozen radioimmunoprecipitation assay buffer (RIPA). After quantification by the BCA method, the standardized protein samples were separated by sodium dodecyl sulfate sodium polyacrylamide gel (SDS PAGE) and transferred to nitrocellulose membrane. Then the membrane was blocked with 5% bovine serum albumin (BSA) at room temperature for 1 h, and then incubated with diluted primary antibody overnight at 4 °C (Abcam, Cambridge, MA). After washing 3 times with Tris-buffered saline (TBST), it was incubated with IgG monoclonal antibody for 12 h at 4 °C. Using INTAS Science Imaging (Göttingen, Germany), the signal development film was incubated with the ECL matrix solution for 1 min for visualization.

### Establishment of OVX Mice Model

Thirty 5-week-old female C57BL/6 mice were divided into sham group, OVX group and OVX + JUG (10 mg/kg) group with10 in each group. Mice in OVX group and OVX + JUG (10 mg/kg) group underwent bilateral ovariectomy and salpingectomy. After OVX surgery, the mice received no treatment and had recovery time of one week. After that, the OVX + JUG (10 mg/kg) group was given a JUG intraperitoneal injection every two days. All animals were sacrificed 9 weeks after the operation. Femur specimens were taken for microCT scanning and histological staining (Zhou et al., 2016).

### Luciferase Reporter Gene Assay

Luciferase Reporter gene assay was used to detect JUG's effect on NF—B or NFATc1. The experimental procedure has been described in previous study (Singh et al., 2012; Zeng et al., 2016). RAW 264.7 cells were stably transfected with either an NF-κB-responsive luciferase construct or an NFATc1-responsive luciferase reporter construct (Wang et al., 2003; van der Kraan et al., 2013). Cells were lyzed and luciferase test substrates (Promega, Madison, WI, USA) were mixed into the samples. BMG Polar Star Optima Luminescent Reader (BMG, Germany) was used to detect fluorescent luminescence. Luciferase activity represented NF—B and NFAT activity.

### Histological Examination and Miro-CT Scanning

The femoral specimens were incubated with 4% paraformaldehyde for 4 days for tissue fixation and with 10% tetracycline-EDTA for 3 weeks for decalcification. The distal femur was cut into 4 mm sections for H&E and TRAP staining. Scanning HE stained sections with Aperio Scanscope, histomorphometric parameters were recorded and analyzed. TRAP staining was utilized to determine the number and morphology of osteoclasts in each section. Micro-CT (Siemens, Germany) was employed to scan 100 slices of each bone growth plate. The image data of bones and trabecular bone were analyzed by Mimics 15.0 software (Matralise, Belgium).

### Statistical Analysis

The results presented are representative of at least three independent experiments and are expressed as mean ± standard error of the mean (SEM). Student's t test and couple ANOVA to a post-hoc test is used to determine the statistical significance between the intervention group and the control group. The one-way analysis of variance model is used to compare multiple groups. The significance level is set to 0.05.

## Results

### Juglanin had Limited Effect on Differentiation and Mineralization of Osteoblasts *in vitro*

**FIGURE 1:**
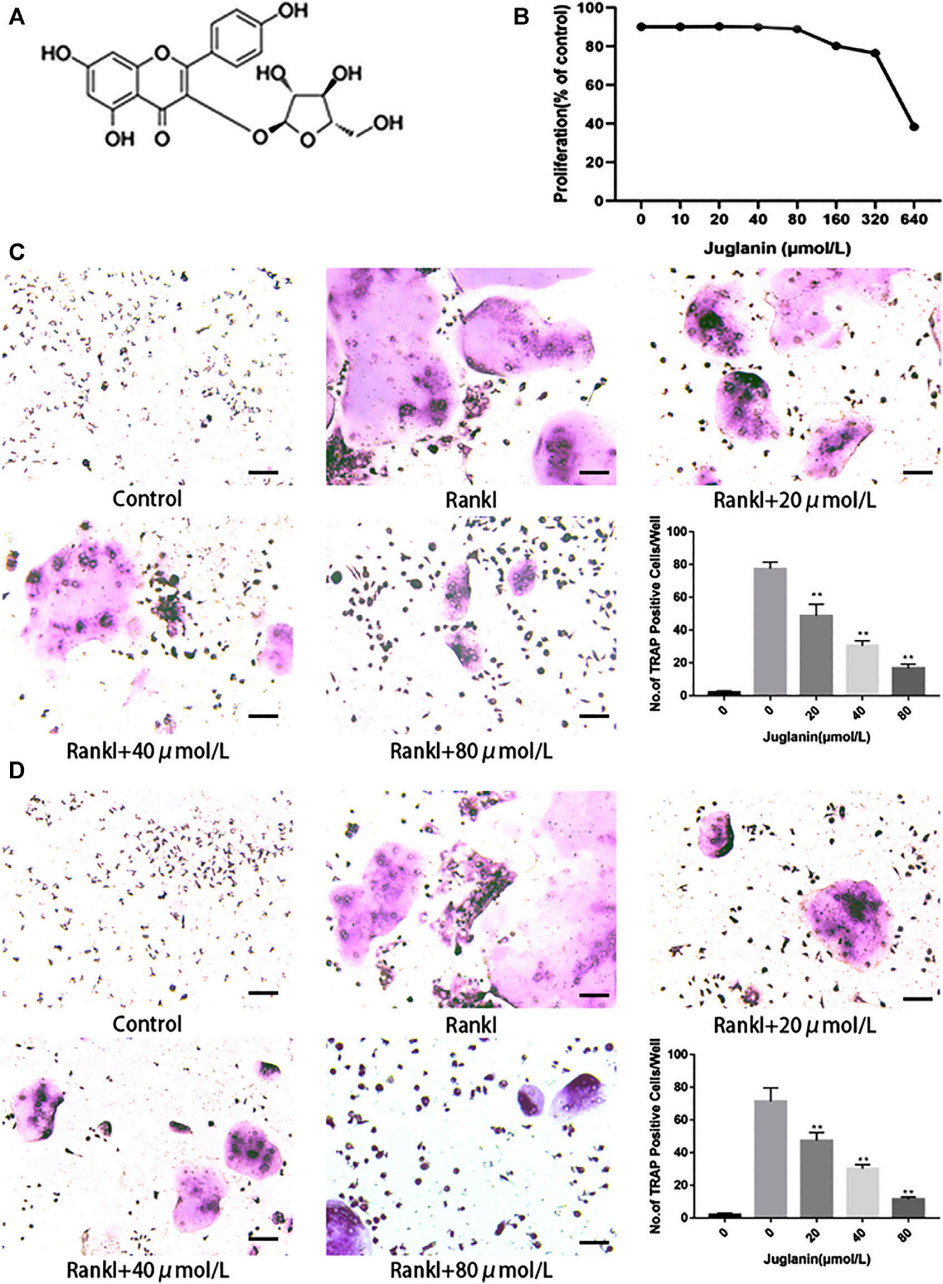
JUG inhibits osteoclastogenesis *in vitro*. All the experiments were performed six times and the average was taken. **(A)** Chemical structure of JUG **(B)** MTT analysis of JUG cytotoxicity in BMSCs. **(C)** Formation of TRAP-positive cells from BMMs and quantification of osteoclast **(D)** Formation of TRAP-positive cells from RAW264.7 cells and quantification of osteoclast. Scale bar 100 μm. Data are presented as the mean ± SEM, *p < 0.05, **p < 0.01 relative to the control group. n = 3

ALP assay and alizarin red staining assay showed that JUG (80 μmol/L) had no inhibitory or promotive effect on BMSCs differentiation (Supplementary Figures S1A,B). Additionally, the cytotoxicity of JUG (10, 20, 40, 80, 160, 320, 640 μmol/L) on BMSCs, as demonstrated in Figure 1B, was evaluated *in vitro* by an MTT assay. No significant difference was observed in terms of cell viability after treatment of 48 h with various concentrations of JUG aforementioned. Thus, the results indicated that JUG had no detrimental impact on two types of cells with concentration no more than 80 μmol/L.

### JUG Negatively Affected RANKL-Induced Osteoclastogenesis in RAW264.7 and BMMs

Two types of cell modes, RAW264.7 and BMMs, were employed to test the impact of Juglanin on osteoclastogenesis *in vitro*. As observed in TRAP staining, the addition of RANKL can effectively induce osteoclast differentiation and formation, whereas the increase of JUG concentration negatively correlated to TRAP-positive multinucleated osteoclasts (>3 nuclei) both in RAW264.7 and BMMs, as demonstrated in Figures 1C,D.

### JUG Inhibited F-Actin Ring Formation

**FIGURE 2:**
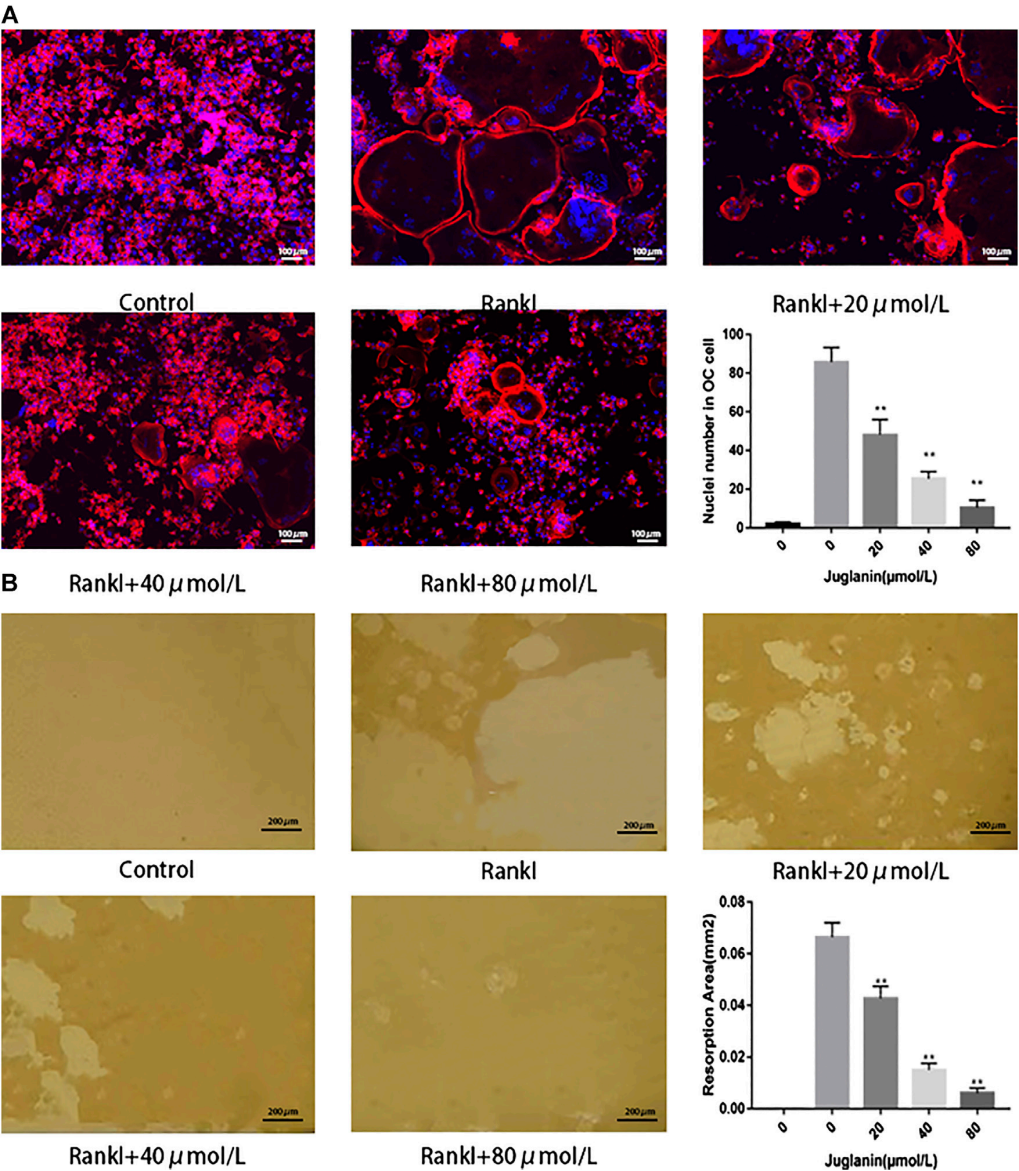
JUG inhibits osteoclasts function. **(A)** F-actin staining of osteoclasts (RANKL-induced BMMs) and quantification of the actin ring **(B)** Pits formation assay of osteoclasts and quantification of resorption area. Scale bar 100 μm. Data are presented as the mean ± SEM, *p < 0.05, **p < 0.01 relative to the control group. n = 3

It was found that rings and multiple intact nuclei emerged after stimulation by RANKL, while the size of F-actin ring and number of nuclei was reduced under JUG treatment (Figure 2A). The results aforementioned indicated that JUG had negative impact on RANKL-induced F-actin formation.

### JUG Suppressed Bone Resorption by Osteoclasts

The capability to absorb bone is the most important function for osteoclasts to exert the physiological role in bone remodeling. As a result, the pit formation assay to determine the resorptive function of osteoclasts was conducted as a golden standard to evaluate the function of osteoclasts. In the present study, osteoclasts were cultured onto bone slices and then intervened with JUG. The results showed that the standardized resorption area was negatively correlated with the concentration of JUG and the control group, with no osteoclasts induced, demonstrated none resorption area, as illustrated in Figure 2B.

### JUG Inhibited Osteoclastogenesis in Early Stage

**FIGURE 3:**
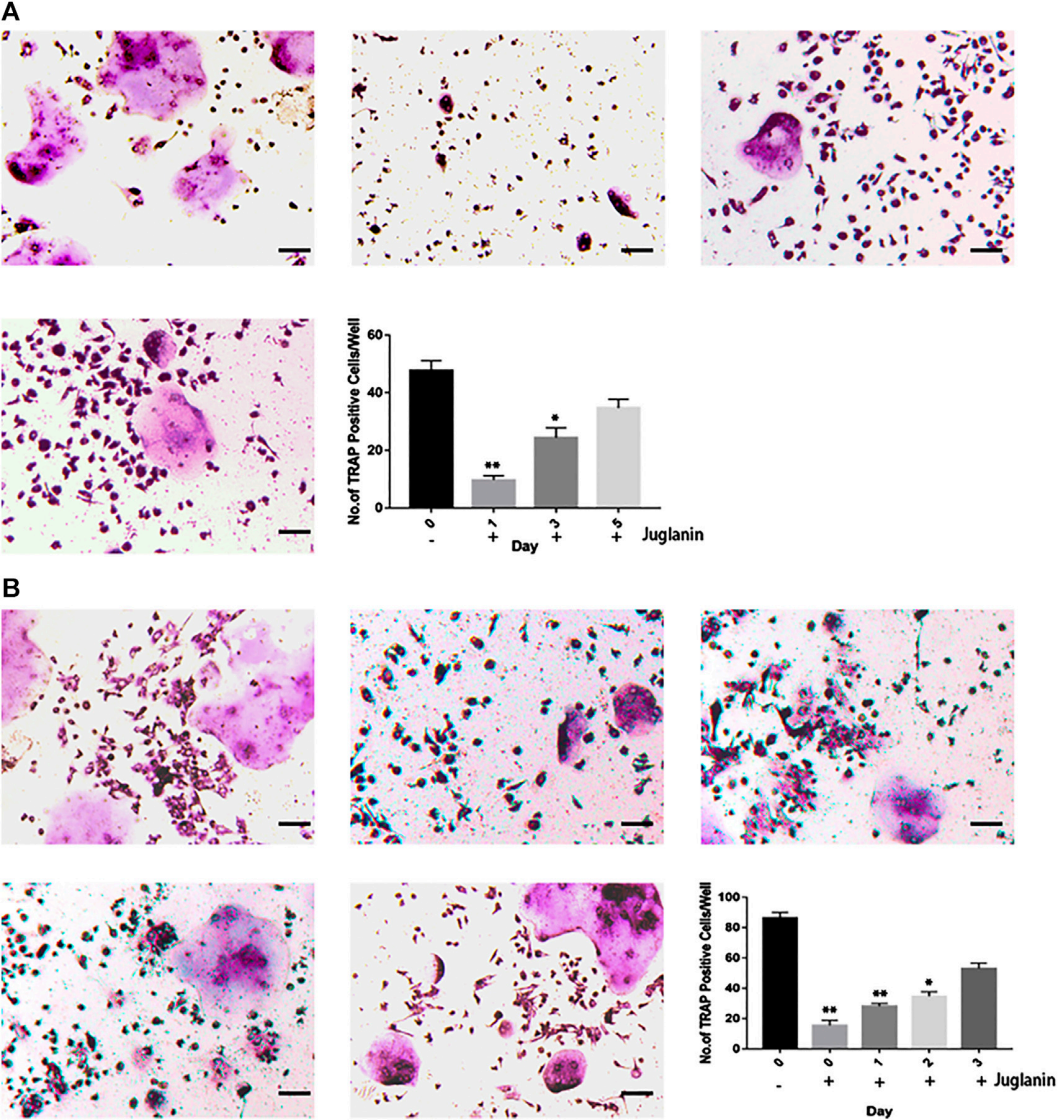
JUG inhibits RANKL-induced osteoclast formation at the early stage. **(A)** Effect of JUG on RANKL-induced BMMs differentiation at different stage **(B)** Effect of JUG on RANKL-induced RAW264.7 cell differentiation at different stages. Scale bar 100 μm. Data are presented as the mean ± SEM, *p < 0.05, **p < 0.01 relative to the control group. n = 3

To identify the timing of osteoclastogenesis aﬀected after the JUG treatment, BMMs were treated with JUG from day 0 to day 5 (Figure 3A) and RAW 264.7 cells from day 0 to day 3 (Figure 3B). The JUG treatment mainly inhibited osteoclast diﬀerentiation on the day1. The results indicated that JUG inhibited the RANKL-mediated osteoclast diﬀerentiation in an early stage.

### Real-Time PCR Demonstrated Decreasing in Osteoclastogenic Gene Expression

**FIGURE 4:**
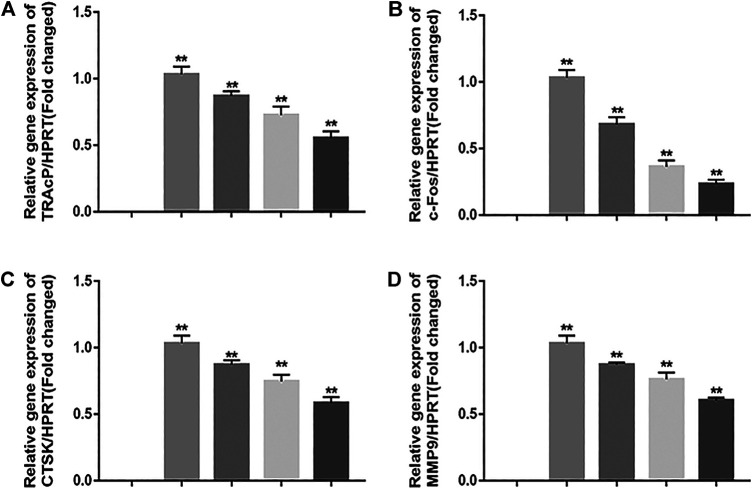
JUG suppresses osteoclastogenic gene expression. **(A–D)** Real-time PCR analysis showing that down-regulates the expression of osteoclastogenic genes c-Fos, TRAcP, MMP9 and CTSK. The expression levels of these genes were normalized to the expression of HPRT. Data are presented as the mean ± SEM, *p < 0.05, **p < 0.01 relative to the control group. n = 3

In order to exam the impact of JUG on osteoclastogenic gene expression, the expression level of c-Fos and other osteoclast-related genes which are essential for osteoclast formation and resorptive function were determined using real-time PCR analysis. It was clearly illustrated that the addition of JUG suppressed the osteoclastogenic gene expression in a dose-dependently, as shown in Figure 4.

### Western Blot Analysis Revealed Reducing Expression of NFATc1 and Related Protein

**FIGURE 5:**
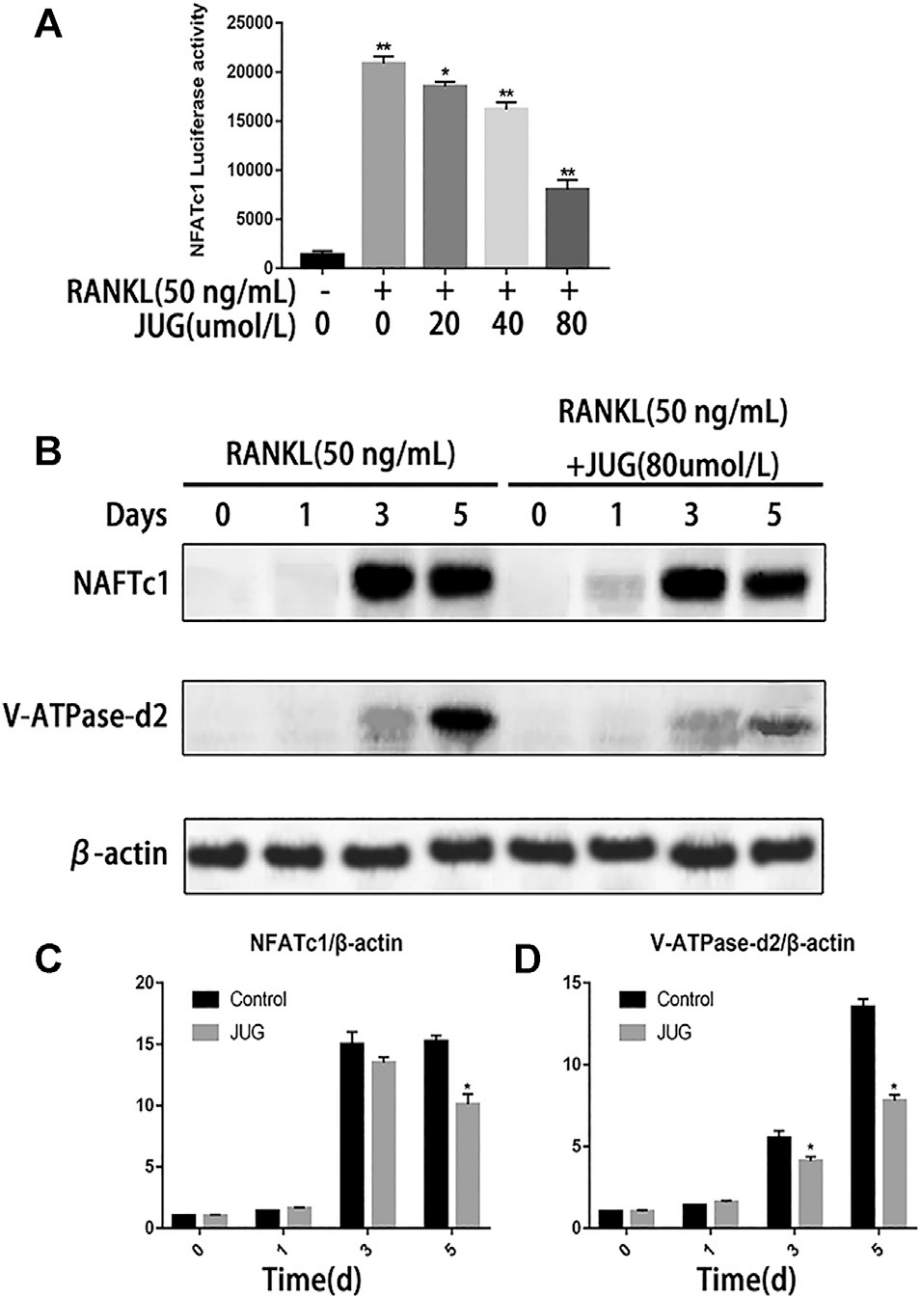
JUG suppresses NFATc1 activity and down-regulates the expression of related protein. **(A)** luciferase reporter gene assay was performed to detect transcriptional activity of NFATc1. **(B)** Representative images of western blots demonstrating the effect of JUG on the expression of NFATc1 and related proteins V-ATPase d2, induced by RANKL on the indicated days. **(C,D)** Relative results were expressed by the ratio of the amount of NFATc1 and related proteins V-ATPase d2 determined by image J. Data are presented as the mean ± SEM, *p < 0.05, **p < 0.01. n = 3

NFATc1 is regarded as the key transcription factors which plays an important part in signal transduction of osteoclastogenesis. Luciferase reporter gene assays were employed to assess NFATc1 transcriptional activity. As demonstrated in Figure 5A, JUG significantly inhibited the NFATc1 expression induced by RANKL. Also, western blot analysis revealed the similar tendency of V-ATPase-d2 expression at day 3 and day 5 which was a downstream protein in NFATc1 pathway as shown in Figure 5B. Thus, the expression of V-ATPase-d2 was down-regulated accordingly (Figure 5C,D).

### JUG Down-Regulation of NF-κB Signaling Pathway

**FIGURE 6:**
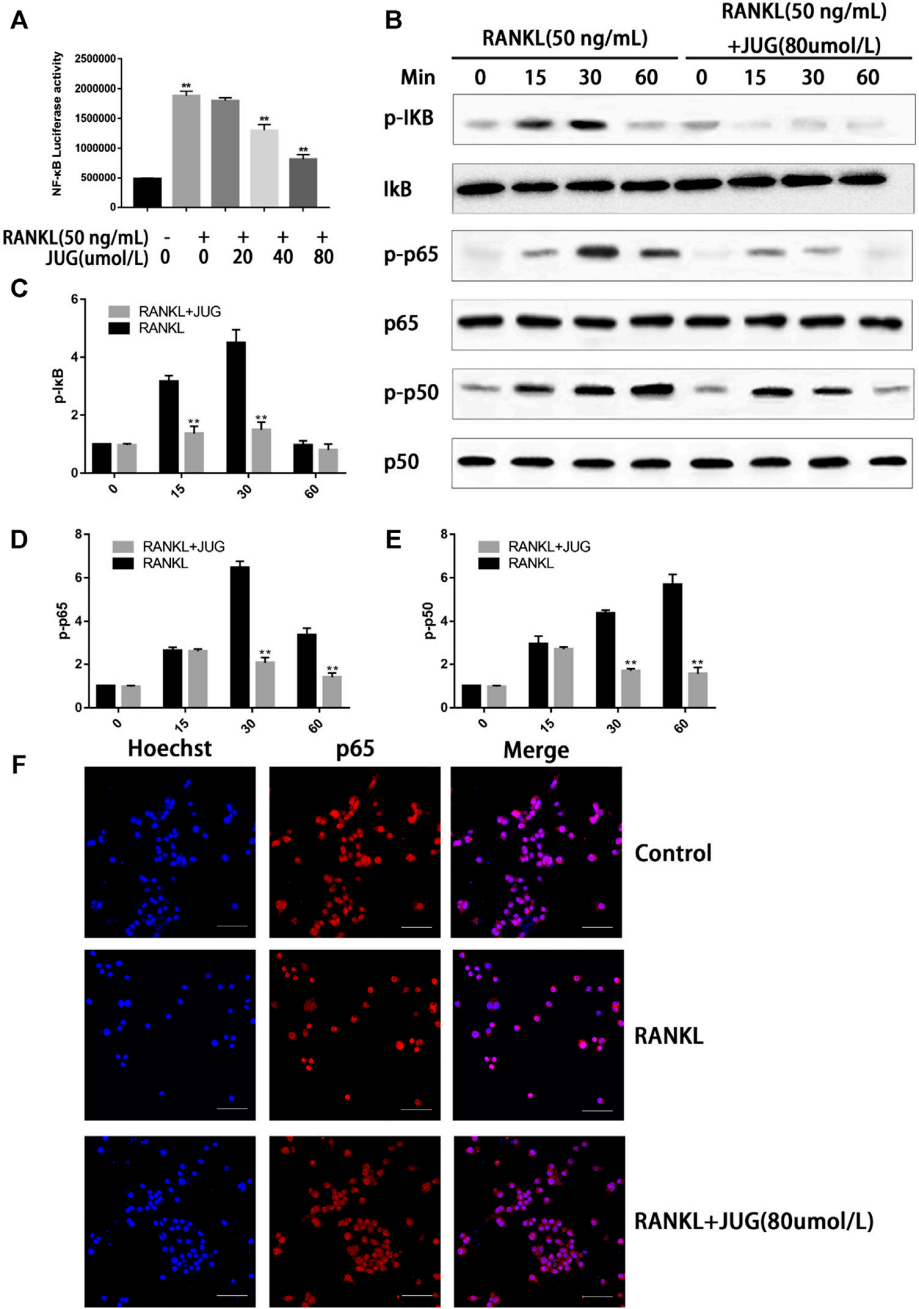
JUG suppresses the NF-κB signaling pathway. The ratio of the fluorescence intensity was quantified by ImageJ software **(A)** luciferase reporter gene assay showing that JUG suppresses the RANKL-induced transcriptional activity of NF-κB. **(B)** Western blot of phosphorylation of IκB, p65, p50 **(D)** Representative image of a western blot demonstrating the effect of JUG on IκB degradation and phosphorylation of p65 and p50 at the indicated times **(F)** JUG inhibits RANKL-induced P65 nuclear translocation. Scale bar 50 μm. Data are presented as the mean ± SEM, *p < 0.05, **p < 0.01. n = 3

Phosphorylation of the NF-kB protein complex under JUG treatment of different concentrations were tested using western blot analysis. It was found that the phosphorylation of IκBα, P50 and P65 was significantly down-regulated 30 min or 60 min after the JUG treatment with the concentration of 80 μmol/L, as demonstrated in Figures 6B–E. With the stimulation of RANKL, p65 was phosphorylated and translocate to the nucleus. Without the RANKL, p65 was mainly located in the cytoplasm (Figure 6F). What’s more, luciferase reporter gene assays revealed similar results as western blot analysis did (Figure 6A).

### Therapeutic Effect of JUG on OVX Mice Model

**FIGURE 7:**
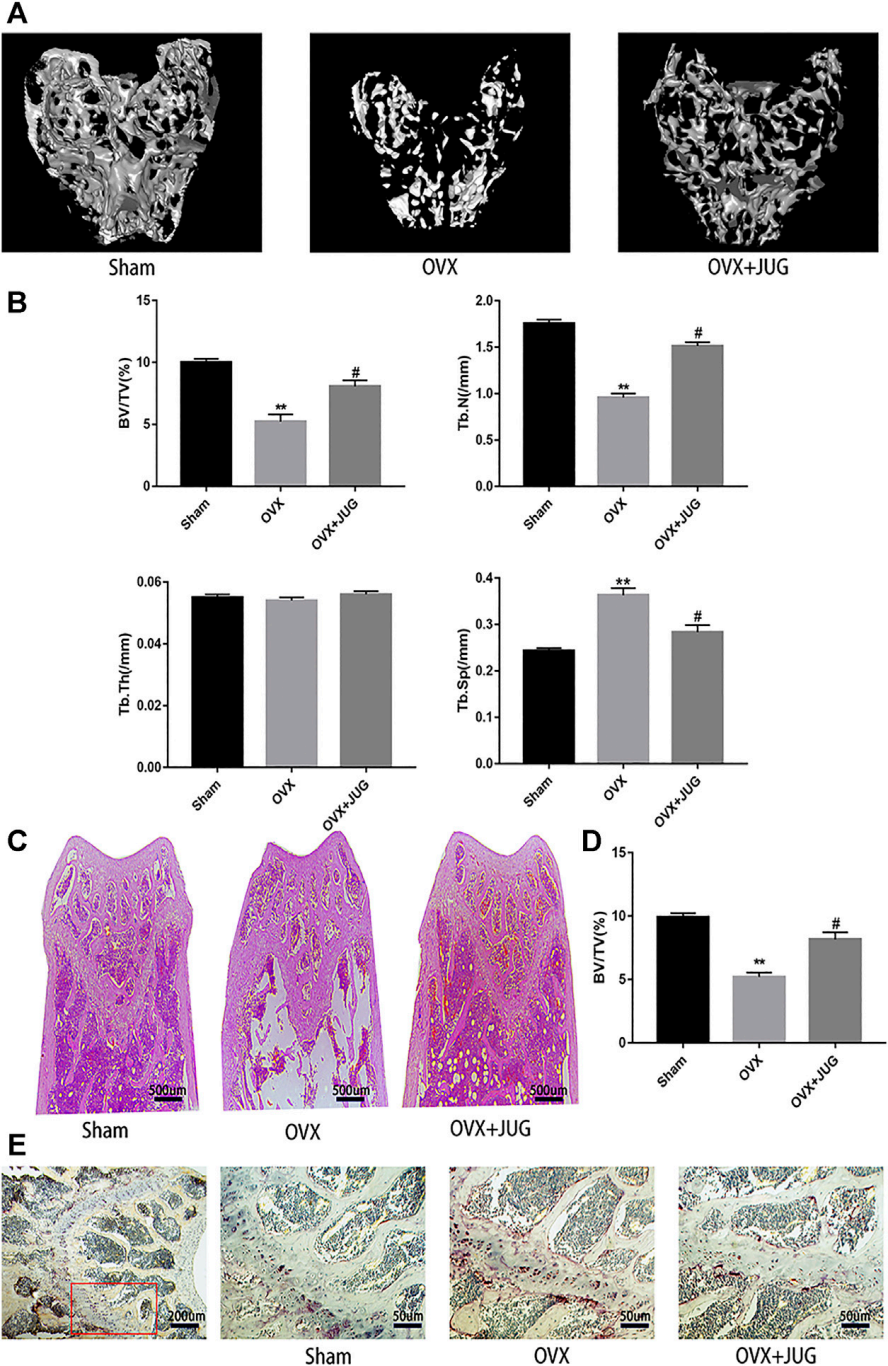
JUG ameliorates ovariectomy-induced bone loss *in vivo*. **(A)** Micro CT analysis of the distal femur from sham, OVX, and OVX + JUG group **(B)** Bone volume per tissue volume (BV/TV), trabecular number (Tb.N), trabecular thickness (Tb.Th) and trabecular separation (Tb.Sp) were analyzed with micro-CT Skyscan CTAn software. **(C–D)** HE staining of distal femoral and quantification of BV/TV **(E)** TRAP-stained histologic distal femur from sham, OVX and OVX + JUG group.

The OVX mice model was built to simulate PMO conditions. Based on the model, the therapeutic effect of JUG to prevent bone loss was studied. H&E (Figure 7C) staining and mica-CT (Figure 7A) were employed to determine changes of trabecular bone structure. In comparison with solvent control group, oral administration of JUG reduced the bone loss, with augmentation in BV/TV and Tb.N and decreasing in Tb. Sp. (Figure 7B,D) However, it is interesting to note that the Tb.Th remained basically the same level among all three groups. Furtherly, TRAP staining (Figure 7E) of decalcified distal femoral sections confirmed the micro-architecture changes of trabecular bone assessed through micro-CT.

## Discussion

Bone metabolism is a physiological process that involves both osteoblasts and osteoclasts. Pathological changes of osteoclasts are commonly seen in osteoporosis diseases. Therefore, it may be a novel and efficient strategy to find natural osteoclasts inhibitors. Many natural substances and their derivatives have potential value to function as osteoclasts inhibitors. Juglanin (JUG) is a natural compound derived from the *crude Polygonum aviculare*. It has been reported the inhibitory effect on inflammation, oxidative stress. In addition, JUG has been explored in the occurrence of human breast cancer through apoptosis, by inducing ROS production in cells. Considering its role. However, there is very little information about the mechanism of JUG on bone activity or related literature reports. As a result, we aim to investigate the potential inhibitory effect of JUG on onsteoclastogenesis in this study.

Pre-experimental results found that 80 μmol/L of JUG can significantly inhibit osteoclast differentiation. After An MTT assay was performed to detect JUG cytotoxicity, we selected 20, 40 and 80 μmol/L dose concentrations for this experiment. The results of this experiment showed that the inhibitory effect of JUG is positively correlated with its concentration. We also found that the effect is mediated through suppressing NF-κB transcription which is a transcription factor in the RANKL/RANK signaling pathway. (Chambers, 2000; Zhao et al., 2007). NF-κB signaling pathway is the most important signaling pathway for osteoclast differentiation and maturation induced by RANKL.

NFATc1 and c-Fos are important transcription factors for osteoclastogenesis. NFATc1 is essential for regulating the process of osteoclast differentiation by controlling osteoclast-related genes, and c-Fos is an important cytosine promoting NFATc1 expression. The results in our study showed that JUG can negatively affect the expression of C-Fos/NFATc1 gene in RANKL-induced osteoclasts. In the later stage of osteoclastogenesis, NFATc1 acts as the terminal transcription factor to regulate the expression of osteoclast-related genes CTSK and TRAP. Previous study reported that over-expression of cathepsin K can lead to loss of bone mass. In this study, JUG can reduce the expression of CtsK gene in osteoclasts induced by RANKL. TRAP is an iron-binding protein, which is highly presented in the generation of osteoclasts and induced osteoclast differentiation. In our study, JUG can also inhibit the expression of TRAP gene in osteoclasts induced by RANKL. Taken together, the binding of RANK to RANKL activates IKK by recruiting the molecule TRAF6. After phosphorylation, IKK degrades IκBα and releases NF-κB (P65) which then enters the nucleus, activates a series of gene expressions that promote osteoclast differentiation, and ultimately promotes osteoclast differentiation. In our research, it was found that the JUG inhibited RANKL induced IκB, P50 and P65 phosphorylation, thus confirming that JUG inhibited osteoclastogenesis through down-regulating NF-κB signaling pathway.

Osteoblast is also a key member in bone metabolism. Therefore, we also studied the effect of JUG on osteoblasts. However, JUG had no inhibitory or promotive effect in terms of ALP staining and Alizarin red staining. In general, JUG has demonstrated no significant effect differentiation and mineralization of osteoblasts.

*In vivo* model showed that JUG prevented bone loss in ovariectomized mice. The number of trap-positive cells around trabecular of distal femur decreased significantly after JUG treatment. The number of osteoclasts in the JUG treatment group was significantly lower. Also, micro-CT showed that after JUG treatment, the percentage of trabecular bone and the number of trabecular bones in OVX mice increased significantly, and the trabecular bone space decreased significantly. Collectively, JUG has demonstrated great potential in preventing osteoporosis.

In conclusion, JUG has negative influence on RANKL induced osteoclast formation *in vitro* and prevent bone loss in mice model. In the molecular mechanism, the inhibition is mediated by NF - *κ* B signaling pathway.

## Data Availability

The original contributions presented in the study are included in the article/Supplementary Material, further inquiries can be directed to the corresponding authors.
